# Residential Exposure to Urban Traffic Is Associated with Increased Carotid Intima-Media Thickness in Children

**DOI:** 10.1155/2015/713540

**Published:** 2015-01-08

**Authors:** Rodrigo X. Armijos, M. Margaret Weigel, Orrin B. Myers, Wen-Whai Li, Marcia Racines, Marianne Berwick

**Affiliations:** ^1^Department of Public Health Sciences, College of Health Sciences, University of Texas at El Paso, El Paso, TX 79968, USA; ^2^Human Immunology, Nutrition and Disease Research Laboratory, College of Health Sciences, University of Texas at El Paso, El Paso, TX 79968, USA; ^3^Center for Health Research and Evaluation, College of Health Sciences, University of Texas at El Paso, El Paso, TX 79968, USA; ^4^Proyecto Prometeo, Secretaría de Educación Superior, Ciencia y Tecnología (SENESCYT), Quito, Ecuador; ^5^Centro de Biomedicina, Facultad de Medicina, Universidad Central del Ecuador, Quito, Ecuador; ^6^Department of Internal Medicine, University of New Mexico, Albuquerque, NM 87131, USA; ^7^Department of Civil Engineering, College of Engineering, University of Texas at El Paso, El Paso, TX 79968, USA

## Abstract

Chronic exposure to urban traffic pollution is documented to promote atherosclerosis in adults but little is known about its potential effects in children. Our study examined the association of long-term exposure to traffic with carotid intima-media thickness (cIMT) in 287 healthy children. Residential proximity and distance-weighted traffic density (DWTD) were used as proximity markers for traffic-related air pollution exposure. The multivariable analyses revealed that children residing <100 meters from the nearest heavily trafficked road had cIMT mean and maximum measurements that were increased by 15% and 11% compared to those living ≥ 200 meters away (*P* = 0.0001). Similar increases in cIMT were identified for children in the highest versus lowest DWTD tertile. Children who resided 100–199 meters from traffic or in the middle DWTD tertile also exhibited increased cIMT but these differences were not statistically significant. No statistically significant differences were identified between residential distance to traffic or DWTD and systemic inflammation indicators (CRP, IL-6). The study results suggest that exposure to urban traffic promotes arterial remodeling in children. This finding is important since even small increases in cIMT over time can potentially lead to earlier progression to atherosclerosis. It is also important because traffic-related pollution is potentially modifiable.

## 1. Background

Cardiovascular disease is a major and growing cause of morbidity, mortality, and disability worldwide [1]. Atherosclerosis, a chronic low-grade inflammatory condition, is the underlying cause of most heart attacks and strokes [2]. The atherosclerotic process, which begins early and progresses over the life course, can be influenced by a number of traditional proinflammatory risk factors (e.g., obesity, physical inactivity, high fat diet, dyslipidemia, hypertension, diabetes, and smoking) and their interactions with genetic susceptibility [2, 3]. Exposure to urban air pollution also has been strongly implicated in promoting atherosclerosis and adverse cardiovascular events. It has been estimated that urban air pollution may be responsible for as much as one-tenth of all global CVD-related deaths and total disability-adjusted life years [1, 3, 4].

Traffic-related air pollution is a major contributor to poor air quality especially in urban centers with high vehicular traffic volume where it can be responsible for as much as 90% of outdoor pollutants present in the local airshed [3, 5]. Vehicular emissions are composed of a complex mixture of CO, CO_2_, NO_*x*_, particulate matter (PM), volatile organic compounds (e.g., benzene, formaldehyde, and acetaldehyde), ozone, and other secondary byproducts (e.g., nitrates and inorganic and organic acids) and other substances. The specific mixture of pollutants associated with mobile source emissions is influenced by fuel type (gasoline, diesel) and quality (e.g., high versus lower sulfur content), vehicle type (heavy- or light-duty), vehicle age, operating and maintenance conditions, exhaust treatment, engine lubricants, wearing of mechanical gears, brake pads, and tires, and road dust present, among other factors [3, 5].

Traffic emissions are a major source of local variability in urban airsheds [5]. Steep spatial gradients exist for traffic-related pollutants such as ultrafine PM (UFP), fine PM (PM_2.5_), coarse PM (PM_10_), CO, and NO_*x*_. The highest exposures occur 50–100 meters from roadways but diminish to approximate background levels by 100–400 meters depending on the type of pollutant and other factors (e.g., meteorological conditions, local topography, and other environmental characteristics) [5–8]. The evidence from diverse toxicological and epidemiologic studies has linked PM, especially PM_2.5_, with many of the adverse cardiovascular effects reported as associated with exposure to traffic emissions [5, 6]. Emerging evidence also suggests that UFP may also be important for reasons of its small particle size which allows for deep penetration into lung tissue, a large surface area which facilitates toxicant absorption, and its inflammatory chemical properties [5, 6].

The evidence from toxicological and epidemiological studies suggests that exposure to PM and other urban air pollutants appears to promote the release of proinflammatory cytokines, reactive oxygen species, and/or endothelial injury and vascular remodeling indicators in adults [5, 9]. It has been hypothesized that repeated inflammatory responses caused by long-term exposure to traffic-related pollutants may promote arterial remodeling and accelerate the atherosclerotic process [5, 10, 11]. Some recent studies have linked long-term exposure to urban traffic pollution with increased arterial calcification, stiffness, and/or carotid intima-media thickness (cIMT) in adults with or without preexisting cardiovascular disease or other chronic conditions [10, 12–15] but others did not [16–18].

Little is known about the potential atherosclerotic effects of traffic-related exposures in children. The special behavioral, anatomical, and physiological characteristics of children increase their potential vulnerability to the negative cardiovascular effects of traffic-related pollutants. They tend to spend more time engaged in outdoor aerobic activities compared to adults. This, along with their smaller lung volumes, higher baseline ventilation rates, tendency to mouth breathe, and other attributes, can expose children to greater pollutant loads compared to older individuals [19, 20]. Several authors have reported that higher exposure to urban air pollutants is associated with increased blood markers of oxidative stress, systemic inflammation, and endothelial dysfunction in children [5, 21–24].

However, only one published study has investigated whether long-term residence in close proximity to urban traffic promotes ultrasound-detectable endothelial remodeling in children. Iannuzzi and associates [25] reported that healthy schoolchildren aged 6–14 years (*n* = 52) who resided 30–300 meters from a major road in a small town on the Amalfi coast of Italy exhibited significantly increased carotid arterial stiffness but not thickness (i.e., cIMT) compared to those living further away, that is, 330–730 and 780–1450 meters. Carotid arterial stiffness is considered to be an early, preclinical atherosclerosis indicator [26]. It is possible that long-term exposure of the children who lived closest to road traffic was sufficient for arterial stiffness to be detected but not enough for cIMT. In addition, it is also possible that the small sample size (*n* = 52) may have reduced the power of the study to detect potential between-group differences in cIMT.

The study of the potential effects of exposure to traffic-generated air pollutants in children is important line of inquiry for the reason that small but progressive increases in fatty streak deposition and arterial thickening over time can lead to earlier progression to clinical disease and premature mortality. The identification of proatherogenic environmental factors such as traffic-related pollution exposure can help to identify underlying mechanisms and target specific strategies to slow atherosclerosis progression and future adverse cardiovascular outcomes. Evidence from such studies can also be used in support of changes in environmental health policy and regulations.

The major objective of the present study was to investigate the hypothesis that living close to heavy urban traffic, a proxy measure for exposure to traffic pollutants, promotes arterial remodeling in healthy children that is detectable as increased cIMT. In addition, we investigated whether close residential proximity to heavy urban traffic is associated with increased systemic inflammation as indicated by C-reactive protein and interleukin-6 (IL-6) serum levels.

## 2. Methods

The study was conducted in the Quito Metropolitan District (QMD), the Ecuadorian capital city. This major urban center of 2.2 million residents is situated in a narrow valley between the eastern and western Andes mountain chains and has an average elevation of 2850 meters (range: 500–4790 m) above sea level [27]. In the last 15 years, population density in the QMD increased from 61 to 91 inhabitants/hectare [27]. Air pollution has become a major public health issue in the QMD [27]. Annual concentrations of all major air pollutants are reported to exceed World Health Organization guidelines excepting ozone [27]. Motor vehicles are a major and growing source of air pollution in the QMD airshed. Private vehicle ownership increased by 29% from 145 vehicles/1000 persons in 2002 to 187/1000 persons in 2008 with an estimated 30,000 additional vehicles added annually [27]. Approximately 8% of circulating buses, trucks, cars, and vans in the QMD are powered by diesel but, by volume, diesel accounts for approximately 35% of the fuel sold in Quito for use in vehicles [28]. The diesel premium (500 ppm) and gasoline (2000 ppm) sold in the QMD for motor vehicle fuel contain the highest sulfur content in Latin America [29]. Mobile sources account for an estimated 98% of CO, 76% of NO_2_, 67% of CO_2_, 53% of NO_*x*_, and 46% of PM annual emissions in the QMD airshed [29]. Nearly one-fourth of QMD residents live within 100 meters of heavily traveled roads and streets [30].

**Figure 1:**
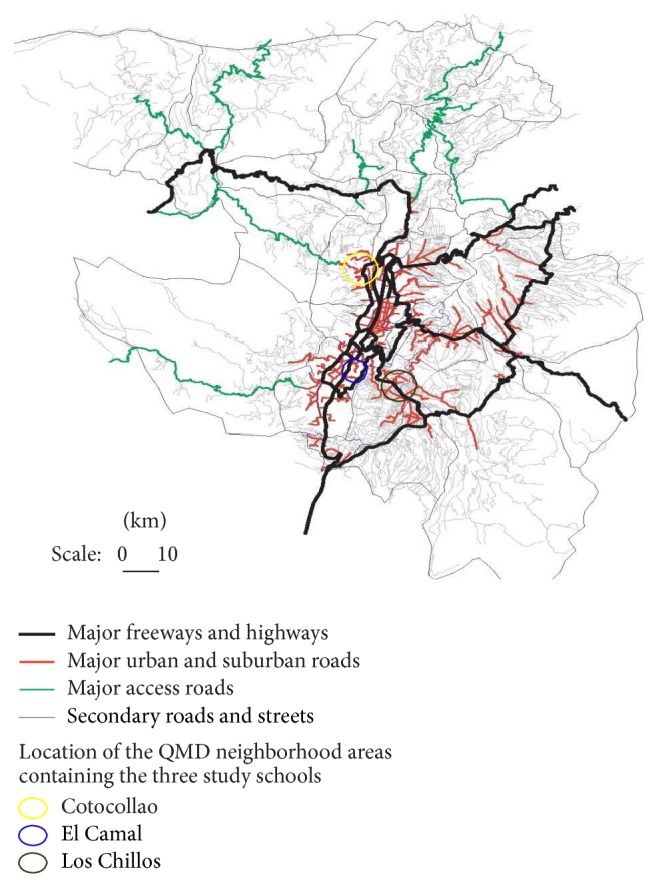
Quito Metropolitan District: location of study neighborhoods and major roadways.

The present study was conducted in three low-income QMD urban neighborhood areas (Figure 1). The three sites were selected based on expected differences concentrations of PM and other ambient air pollutants collected from QMD central air monitors during the 12 months prior to the start of the study [Dr. Patricia Eschanique, Director, Direccion Metropolitano Ambiental, personal communication]. These were El Camal (most heavily polluted), Cotocollao (medium polluted), and Los Chillos (least polluted). The data used for the present analysis were collected from April to June 2010. One public elementary school in the neighborhood areas was identified based on its close proximity to a QMD central air monitor (≤5 km) and estimated air pollutant concentrations. The investigators first held meetings with school officials, teachers, students, and their parents to explain the purpose of the study and answer questions. A computerized random numbers list was generated to select ~100 child participants from each school site.

Prospective participants from the three elementary schools were eligible for study inclusion if they were aged 7–12 years, were healthy, and resided within five miles of a QMD central air monitor in the same neighborhood. They were excluded from participation if they had showed clinical evidence of or had a positive history for cardiovascular disease, dyslipidemia, diabetes, or other serious chronic or infectious conditions (e.g., TB, HIV/AIDS) or lived in a home with indoor smokers or if another child from their household was already enrolled in the study. Thirteen prospective child participants were excluded from the study because they showed evidence of a chronic disease (*n* = 1), had indoor smokers present in their household (*n* = 6), or had siblings already enrolled in the study (*n* = 4) or if their parents declined to complete the informed consent process (*n* = 2).

The study protocol was approved by the University of Texas at El Paso and the University of Central Ecuador Biomedical Ethics institutional review boards. Verbal and written informed assent was obtained from the child participants and informed consent for their child to participate in the study was obtained from parents/guardians.

Trained interviewers who were native (Ecuadorian) Spanish speakers collected detailed data on child participants, household, and neighborhood characteristics during face-to-face interviews with participants parents/guardians. The sociodemographic characteristics data included child age, sex, ethnicity, parental occupation, marital status, residential history, monthly household and* per capita* income, and family size and composition. Household and neighborhood environmental characteristics data included child residential history, home construction, household operation behaviors involving cooking, heating, trash burning, and other pollutant sources (e.g., candles, incense), home ventilation practices, household hobbies/recreational activities (e.g., woodworking, metal work, and plastic models), and any outdoor smoking behavior by household members. Detailed data also were collected on the source and location of local neighborhood pollutant sources (e.g., street food stands, LPG depositories, gasoline stations, car repair shops, and factories). Child and family health histories were obtained from parents/guardians. These focused on cardiovascular and other serious chronic (e.g., diabetes) and infectious conditions (e.g., TB). Data collected on child activity patterns included the timing, physical location, and type of indoor and outdoor activities. These were obtained by physical activity recalls administered to the child participants and their parents/guardians.

Weight, height, and midupper arm circumference measurements were obtained for each child participant. Measurements were performed without shoes or headgear in a hospital gown. Weight was measured to the nearest kilogram on a calibrated electronic balance (Detecto, IN). A portable stadiometer (Seca, Chino, CA) was used to measure child height in meters. The weight and height measurements were used to calculate body mass index. Midupper arm circumference was measured to the nearest centimeter with a semiflexible anthropometric measuring tape (Seca, Chino, CA).

A 12-hour fasting blood sample (7 mL) was donated by the child participants for the purpose of measuring total cholesterol, HDL, triglyceride, and glucose with the Modular P-800 chemistry analyzer (Roche Diagnostics, IN). A portion of the blood sample was also used to analyze high sensitivity C-reactive protein and IL-6 (Roche Diagnostics, IN). All samples were analyzed in the Biomedical Research Institute at the Central University of Ecuador medical campus.

An experienced pediatric cardiologist (IZ) performed all cIMT ultrasound studies. He was blinded to the child participant residence and exposure characteristics. The cIMT studies were performed using the MicroMaxx 3.4.1 high-resolution M-mode digital ultrasound portable system linked to a 5–10 MHz multifrequency high-resolution linear transducer (Sonosite, Bothwell, WA). Participants were placed in a supine position with the head extended and slightly rotated to the opposite side using a 45-degree head block [31]. The cIMT measurements were performed after participant had rested quietly for 10–15 minutes. Semiautomatic measurements of mean and maximum CIMT were made using SonoCalc version 4.1 edge detection software (Sonosite, Bothwell, WA). Thickness was assessed as both the mean and maximum of three predefined angles (anterior, lateral, and posterior) capturing the media-adventitia interface of the near and far arterial walls, 1 cm proximal to the bulb from both right and left carotid arteries.

The pediatric cardiologist also performed the blood pressure measurements using a calibrated manual sphygmomanometer with adjustable pediatric cuff following the American Heart Association recommendations [32]. Three readings were taken (2-minute intervals) and the average was recorded as mean systolic and diastolic blood pressure. These measurements were used to calculate mean arterial pressure (MAP = [(2 × diastolic blood pressure) + systolic blood pressure]/3.

We obtained data on the current address and residential history of each child participant from their parents. The unique residential addresses of participants were physically verified by the study team and subsequently geocoded into latitude and longitude coordinates. The major traffic arteries for each Quito neighborhood zone were identified based on traffic volume studies collected by the QMD Department of Transportation and Public Works [33]. Google Earth (Mountain View, CA) was used to obtain *X* and *Y* coordinates for longitude/latitude orientation and straight line distance (in meters) from each unique residence to the nearest major traffic artery in each zone.

We used two proxy measures to assess child residential exposure to traffic emissions. The first was residential proximity to the nearest heavily trafficked road, defined as having a daily volume of ≥10,000 vehicles/day. We performed sensitivity analyses for both cIMT mean and cIMT maximum to determine the optimum roadway distance cut-off point. Based on the sensitivity analysis results, residential proximity in this study was defined as <100, 100–199, and ≥200 meters from the nearest heavily trafficked road (≥10,000 vehicles/day).

We also calculated a distance-weighted traffic density (DWTD) value for each child participant. We constructed a 754.5 ft. (230 m) radius buffer around the residence of each participant and used a basic model to estimate motor vehicle exhaust dispersion from roadways. The DWTD model is based on the assumption that vehicular exhaust emissions follow a Gaussian distribution in which 96% of pollutants emitted undergo dispersion in vertical and horizontal directions at a distance of approximately 500 feet (152.4 m) from the center of the road [34]. Published studies have confirmed that dispersal of vehicle exhaust pollutants approximate the 500 ft, distance from roadways although exact dispersion distances varied by study site and pollutant type [5]. In the DWTD model used for the study, *d* represents the shortest distance from the child participant's residence to each major roadway within the buffer we constructed. *y* represents the value that was used in the model to weigh the vehicle flow obtained for each road within this area:

(1) $$\begin{array}{c} y=\frac{1}{0.4\sqrt{2\pi }}\exp\left[\frac{0.5{\left(d/500\right)}^{2}}{0.4^{2}}\right]. \end{array}$$

Data on vehicle flow for the roadways of interest in the study were obtained from traffic volume studies conducted by the QMD Department of Transport and Public Works [33].

Historical data from QMD central air monitoring stations were used to obtain estimates on one-year and five-year annual mean PM_2.5_ and ozone background concentrations for each of the three study neighborhoods for the respective 12- and 60-month periods immediately prior to cIMT measurement in the children. We were not able to calculate lifetime PM_2.5_ and ozone exposure due to the lack of reliable QMD central air monitor data prior to 2005.

Since all child residences (and schools) were located within close distance (i.e., 5 kilometers) from one of the three QMD central monitors, we used these data from these to estimate annual residential exposure. We estimated childhood background exposures corresponding to the prior five-year period (2005–2010) by averaging exposures across the relevant residential histories for those time periods. We used published QMD central air monitor station data to estimate annual mean PM_2.5_ and ozone background concentrations for the study children. In the event that they had lived in multiple residences during the exposure periods of interest, time was split by weighting the location-specific exposure estimates according to duration of residence spent at that location.

Historical data on background annual PM_2.5_ for 2005–2010 were available from QMD central air monitors for the El Camal and Cotocollao but not the Los Chillos neighborhoods. However, annual PM_10_ data were available for Los Chillos and Cotocollao but not El Camal. To address this challenge, it was decided to estimate annual background PM_2.5_ for the Los Chillos site using the PM_2.5_/PM_10_ ratio method [35] in which we used a factor of 0.57 (mean annual PM_2.5_/PM_10_ ratio reported for QMD for 2005–2010). Data were not available on CO, NO_*x*_, or SO_4_ from all three central air monitoring sites and there was no way to estimate these so we did not include these background pollutants in the analyses.

### 2.1. Data Analysis

Descriptive data are presented as frequency (%) or means ± S.D. Initial bivariate analyses were conducted to compare differences in proportions using *χ* ^2^ or Fisher's exact test, as appropriate. These initial analyses analyzed mean differences using independent *t*-tests or one-way analysis of variance to examine between-group differences in continuous variables, as appropriate. Nonnormally distributed variables underwent logarithmic transformation prior to analysis. Statistical significance was defined as *P* < 0.05 and all hypothesis tests were 2-sided. Generalized linear models were constructed to examine the association of residential distance to traffic and DWTD with child cIMT mean and cIMT maximum. Potential confounders selected for* a priori* inclusion in the statistical models were those previously reported to be associated with cIMT in healthy children (i.e., age, sex, BMI, and blood pressure) or documented risk factors for atherosclerosis such as positive family CVD history, blood cholesterol, systemic inflammation (hsCRP, IL-6), neighborhood background air pollutants (PM_2.5_, O_3_), and presence of an outside tobacco smoker living in the household. In addition, the analyses also adjusted for other exposure variables identified in the initial analyses which altered effect estimates by >10%, that is, exterior residential window ventilation practices and time spent outdoors.

## 3. Results

Of the 302 children who were initially enrolled in the study, 15 were excluded from the present analyses because they failed to show up for their first scheduled visit (*n* = 3) or subsequent cIMT ultrasound exams (*n* = 12). However, their sociodemographic characteristics were comparable to those of the 287 children who completed the study including age (8.6 ± 1.4 yrs versus 8.9 ± 1.4 yrs, *P* = 0.42), male gender (66.7% versus 55.1%, *P* = 0.43), ethnic minority (0% versus 3.8%, *P* = 0.49), average time of residence in the current neighborhood (8.1 ± 1.7 yrs versus 8.4 ± 2.1 yrs, *P* = 0.6), and monthly household* per capita* income ($73 ± 55 versus $83 ± 49, *P* = 0.5).

### 3.1. Participant Characteristics

Table 1 displays the selected sociodemographic, nutrition, and health characteristics of the child participants and their households stratified by residential distance to traffic. All 287 of the study children were from low-income, working class households and attended one of three local public elementary schools located in Cotocollao (100; 34%), El Camal (94; 32.8%), and Los Chillos (93; 32.4%). Three-fourths of the child participants had a normal weight for age and sex; one-fifth were overweight or obese. One-quarter had a positive family history for hypertension and 15–20% had a positive family history for diabetes, hypercholesterolemia, or heart attack/stroke. As Table 1 also shows, although the proportion of overweight/obesity was marginally higher among children who resided the furthest away from traffic (>200 meters), no other statistically significant between-group differences were identified regarding participant sociodemographic and health-related characteristics including CRP and IL-6. The same general associations shown in Table 1 for residential proximity to traffic were also identified for the characteristics stratified by DWTD tertile (data not shown).

Table 2 reports on the exposure-related characteristics of the child participants. All children had lived in their current QMD neighborhood during the past 12 months and most (87.8%; 252) were lifetime residents of the same. Another 8.4% (24) had previously resided in another QMD neighborhood and 3.8% (11) had lived in another city. Forty-four percent of the children lived less than 100 meters from the nearest road with a daily traffic volume of 10,000 or more vehicles/day. As Table 2 indicates, no statistically significant differences were identified among the three groups regarding the reported residential and individual exposure characteristics with two exceptions. The mean altitude of participant homes was 141 and 95 meters lower in Los Chillos compared to El Camal and Cotocollao, respectively. In addition, the daily vehicular volume was increased among children who resided 100–199 meters from the nearest heavily trafficked road with >10,000 vehicles/day compared to those who lived further away. Table 2 also shows that the three residential distance groups were similar regarding the presence of air pollutant-emitting industries in their neighborhood and background concentrations of ozone. Annual 1-year and 5-year PM_2.5_ background concentrations were higher among children who lived less than 200 meters from the nearest heavily trafficked road.

### 3.2. Association of Traffic Exposure Indicators with cIMT

Table 3 shows the results of both the unadjusted and adjusted statistical models examining the association of the two traffic exposure indicators, residential distance to traffic and distance-weighted traffic density, with the cIMT mean and maximum ultrasound measurements. The unadjusted analysis results revealed that close residential proximity to the nearest heavily trafficked road was positively associated with higher cIMT mean and maximum measurements. Specifically, children who lived less than 100 meters from traffic had respective average cIMT mean and maximum measurements that were 11–15% greater compared to those who resided ≥200 meters away from traffic. Children living 100–199 meters away also had greater mean and maximum cIMT measurements compared to those residing ≥200 meters away although this difference was not statistically significant.

A similar pattern was seen for distance-weighted traffic density. Children exposed to the highest DWTD tertile had respective cIMT mean and maximum measurements that were increased by 12% and 10% compared to those living in the lowest tertile. Those in the middle tertile also had greater average cIMT mean and maximum values compared to those residing ≥200 meters away but the difference was not statistically significant (Table 3).

Adjustment for covariates reported to influence cIMT in healthy children, that is, age, sex, BMI, and blood pressure, cardiovascular risk factors, that is, total cholesterol and family CVD history, or systemic inflammation markers such as hsCRP and IL-6 (Model 1) resulted in only minimal changes in model estimates for both residential proximity to traffic and DWTD analyses.

The final cIMT mean and maximum models were adjusted for exposure variables (Table 3). The exposure variables included 5-year neighborhood background annual PM_2.5_ and ozone, altitude of home residence, presence of an outside smoker in the home, time spent outdoors, and time that exterior windows were left open (Model 2). The latter two variables were added to the model as preliminary analyses indicated that child participants who spent an average of 240 minutes/day outdoors (e.g., engaged in play, walking to and from school, and running errands) had significantly increased average cIMT mean (3.9 *μ*m ± 0.07 versus 3.7 *μ*m ± 0.07; *F* = 5.7; *P* = 0.017) and maximum measurements (5.4 *μ*m ± 0.06 versus 5.2 *μ*m ± 0.07; *F* = 5.8; *P* = 0.016). Likewise, children who lived in households where outside windows were open for an average of 60 minutes or more per day also exhibited significantly increased CIMT mean (3.8 *μ*m ± 0.07 versus 3.4 *μ*m ± 0.08; *F* = 7.5; *P* = 0.007) and maximum measurements (5.3 *μ*m ± 0.07 versus 4.8 *μ*m ± 0.09; *F* = 8.7; *P* = 0.003). However, the model estimates changed a little even with the addition of the exposure covariates (Table 3).

## 4. Discussion

This study is the first to demonstrate that long-term residential proximity (<100 meters) to highly trafficked roadways, a proxy measure for traffic-related pollution, promotes ultrasound-detectable arterial remodeling in children. Our study findings are strengthened by factors such as the apparent dose-response effect of increasing traffic-related exposure and cIMT, control for covariates reported to specifically influence cIMT in healthy children and other documented atherosclerosis risk factors, and other individual, residential, and neighborhood exposures. The study adds to the growing body of evidence documenting the proatherogenic effects of human exposure to traffic-related air pollution. This is important because even small but progressive increases in arterial thickening that begins early in childhood due to air pollutant exposures could potentially lead to earlier progression to clinical atherosclerosis and its associated sequelae.

An estimated 600 million urban inhabitants worldwide are currently exposed to high levels of traffic-related air pollution [36]. A substantial proportion of these, including children, live within 100 meters of heavily trafficked major roadways in cities [5]. The proportion of children and other exposed persons is expected to rise over the next several decades as the proportion of humans living in cities increases.

Only one other published study has reported on the relationship between long-term residential proximity to traffic and ultrasound-detectable vascular remodeling markers in children. Different from our results, Iannuzzi and colleagues [25] reported that close residential proximity to traffic was not associated with significantly increased cIMT thickness in similarly aged, healthy Italian children. However, they did report that it was associated with another early, preclinical atherosclerosis marker which we did measure in our study, that is, increased stiffness of the carotid artery. Increased arterial stiffness is reported to be detectable earlier than arterial thickness in children with cardiovascular risk factors [37]. We did not measure arterial stiffness in the present study so we cannot compare this result. Although methodological differences (i.e., sample size, model covariates, distance proxy categories, and traffic distance categories) may partially explain the discrepancy in findings, it also seems likely that differences in the level of exposure to traffic pollutants may also be responsible. Our study was conducted in the Quito Metropolitan District (QMD), a large major urban center with 2.2 million residents, whereas that of Iannuzzi et al. took place in a small Italian coastal town,* Vietri sul Mare* (8,600 residents) [25]. At the time of the study, the QMD had 414,788 circulating vehicles [38] and the volume of traffic measured on major roadways near the residences of the study participants averaged 27,354 ± 16,060 vehicles/day (range: 10,000–66,484 vehicles/day) [38]. We were unable to locate data on vehicular traffic for* Vietri sul Mare* but it seems likely that, due to its small size and location, the amount of traffic-related air pollution produced by traffic on the relatively small coastal roadway was several orders of magnitude lower compared to our major urban study site. It is also possible that the higher altitude of the Ecuadorian study site (2820 m) could have increased the exposure of children to traffic-related air pollutants compared to that of the Italian location (240 m). At this altitude, combustion engines work less efficiently and emit more pollution due to reduced atmospheric oxygen content. In addition, children living at high altitude have higher baseline respiratory rates due to the lower oxygen content of the air which may have also increased their exposure to any traffic-related pollutants present.

Systemic inflammation is one of several complementary biological mechanisms hypothesized to explain the reported association between exposure to traffic-generated and other urban air pollutants, especially PM, and cardiovascular health indicators including atherosclerosis [4, 5, 11]. However, the findings from epidemiological studies are inconsistent regarding whether exposure to specific PM fractions and other common urban air pollutants is associated with increased systemic inflammation as measured by blood CRP or IL-6 [9, 10, 13, 39–46]. Relatively few studies have investigated the association of specific residential indicators of exposure to traffic emissions with these two systemic inflammation indicators. In our study, close residential proximity to traffic or distance-weighted traffic density was not associated with either inflammation indicator. Our results are consistent with several studies conducted in adults [45, 47] and children [25, 48] which reported a lack of association with CRP or IL-6. However, they differ from findings from two other studies indicating that CRP was positively associated with residential traffic proximity and density indicators in adults [49, 50], especially long-term residential proximity [51]. The reason for the reported discrepancies among studies could be due to differences in the amount and length of exposure to traffic-related air pollution or other methodological or population differences related to age, BMI, and other attributes. It is also possible that since both CRP and IL-6 are nonspecific indicators of systemic inflammation, infections, injury, obesity or proinflammatory chronic conditions, or other sources of inflammation may have accentuated or masked the effects of traffic pollutant exposure. This is especially likely in our Ecuadorian population where clinical and subclinical respiratory and gastrointestinal and other infections are common.

The potential limitations of our study should be considered when interpreting its results. One of these is that the cross-sectional design of the study limits the ability to prove causation between traffic-related exposure and cIMT. As in any epidemiological study, it is possible that residual confounding could have occurred. However, since the study was specifically designed to investigate atherosclerosis risk factors, we were able to control for multiple potential health, exposure, and sociodemographic confounders although we did not control for residential exposure traffic noise. Exposure misclassification could represent another potential source of error. We used proxy indicators of exposures at child residences instead of individual measures of PM and other traffic-related pollutants reported to promote atherogenesis. One of the two proxies used in our study for exposure to traffic-related air pollution was straight line distance from participant residence to the nearest major traffic artery. However, residential distance to a major roadway is reported to be a useful proxy measure for long-term exposure to traffic-related pollutants when assessed on a very small scale such as in our study [12]. In addition, we used a second proxy measure, distance-weighted traffic density (DWTD), which combines data on residential proximity to traffic with vehicular traffic volume to estimate exposure to air pollutants. It is also possible that other unmeasured factors also could have affected residential exposure to traffic-related pollution levels including wind flow, side of street, precipitation, urban canyons, and other physical structures. Moreover, assessment of residential exposure may not be an accurate reflection of individual exposure levels. However, we also controlled for child outdoor activity and outdoor air filtration into residential indoor spaces.

In conclusion, the results of our study indicated that childhood exposure to traffic-generated air pollutants, as indicated by residential proximity and traffic density proxy markers, promotes atherogenesis in healthy elementary school age children. The present work provides support for the hypothesis that living close to heavily trafficked roadways is detrimental to cardiovascular health. Prospective cohort studies are needed to confirm our findings and identify the major traffic pollutants responsible since traffic-related pollution is potentially modifiable through changes in urban planning policy, strengthening and enforcement of clean air laws, use of cleaner technology, and other methods.

## Figures and Tables

**Table 1:** Selected child and household sociodemographic and health characteristics stratified by residential proximity to traffic (*n* = 287).

Characteristics	Residential proximity to traffic		
	<100 m (*n* = 126)	100–199 m (*n* = 91)	≥200 m (*n* = 70)
	Mean ± S.D.	Mean ± S.D.	Mean ± S.D.
	Number (%)	Number (%)	Number (%)
Child and household sociodemographic characteristics			
Child age (yrs)	9.0 ± 1.5	9.0 ± 1.5	8.8 ± 1.3
Child gender (male)	58 (46.0)	41 (45.1)	30 (42.9)
Child ethnicity (mestizo)	119 (94.4)	87 (95.6)	70 (100.0)
Household size (number of members)	5.1 ± 1.6	5.1 ± 2.0	4.9 ± 1.6
Monthly household *per capita* income (US$)	83.0 ± 49	87.0 ± 53	80.0 ± 49
Child lives in single parent home	20 (15.9)	12 (13.2)	14 (20.0)
Mother's education (years completed)	8.5 ± 4	7.5 ± 3	7.3 ± 3
Paternal education (years completed)	9.3 ± 3	8.2 ± 3	7.8 ± 3

Child nutrition and health characteristics			
Underweight for age-and-sex	11 (8.7)	6 (6.6)	2 (2.9)
Normal weight for age-and-sex	93 (73.8)	69 (75.8)	43 (61.4)
Overweight/obese	22 (17.5)	16 (17.6)	25 (35.7)^*^
Midupper arm circumference (cm)	19 ± 2.2	19 ± 2.3	19 ± 2.6
Blood pressure			
Systolic BP (mmHg)	93 ± 8	93 ± 8	93 ± 8
Diastolic blood pressure (mmHg)	65 ± 6	65 ± 6	64 ± 6
Mean arterial pressure	74 ± 6	74 ± 6	74 ± 5
Fasting blood lipid profile			
Total blood cholesterol (mg/dL)	175 ± 28	169 ± 28	180 ± 35
Low-density lipoprotein (mg/dL)	94 ± 21	90 ± 21	96 ± 25
High-density lipoprotein (mg/dL)	58 ± 12	56 ± 13	56 ± 13
Triglycerides (mg/dL)	81 ± 34	87 ± 74	91 ± 43
Fasting blood glucose (mg/dL)	88 ± 10	86 ± 9	87 ± 9
High sensitivity C-reactive protein (mg/L)	1.1 ± 2.0	2.8 ± 7.8	1.7 ± 4.5
Interleukin-6 (pg/L)	7.3 ± 4.9	6.0 ± 3.6	6.3 ± 4.7

Family health history			
Heart attack or stroke	19 (15.1)	18 (19.8)	10 (14.3)
Hypertension	39 (30.2)	21 (23.1)	14 (20.0)
Hypercholesterolemia	20 (15.9)	10 (11.0)	13 (18.6)
Type 2 diabetes	28 (22.2)	18 (19.8)	12 (17.1)

^*^ *P* = 0.04.

**Table 2:** Selected residential, individual, and neighborhood exposure indictors stratified by residential proximity to trafficked roads with >10,000/vehicles/day (*n* = 287).

Characteristics	Residential proximity to traffic		
	<100 m (*n* = 126)	100–199 m (*n* = 91)	≥200 m (*n* = 70)
	Mean ± S.D.	Mean ± S.D.	Mean ± S.D.
	Number (%)	Number (%)	Number (%)
Residential and individual exposure characteristics			
Residence in current neighborhood ≥12 months	126 (100.0)	91 (100.0)	70 (100.0)
Years of residence in current neighborhood	8.4 ± 2.2	8.5 ± 2.1	8.5 ± 1.1
Altitude of child residence (meters above sea level)	2879 ± 189	2833 ± 192	2738 ± 247^*^
Home size (m^3^)	72.0 ± 66	63.0 ± 40	66.0 ± 42
Home construction: cement block	105 (83.3)	73 (80.2)	56 (80.0)
Number of functional exterior windows in home	2.6 ± 1.7	2.2 ± 1.8	2.5 ± 1.7
Exterior windows opened >60 min/day	115 (91.3)	84 (92.3)	64 (91.4)
Cooking fuel: bottled liquefied propane gas (LPG)	126 (100.0)	91 (100.0)	70 (100.0)
Residential trash burning next to home (yes)	13 (10.3)	12 (13.2)	10 (14.3)
Outdoor smoker living in household (yes)	27 (21.4)	19 (20.9)	9 (12.9)
Candle or incense burning in home (yes)	25 (19.8)	15 (16.5)	9 (12.9)
Average time child spent outdoors (hrs/day)	3.1 ± 1.3	3.1 ± 1.4	3.5 ± 1.1
High child outdoor exposure (≥4 hrs/day)	28 (22.2)	22 (24.2)	22 (31.4)

Neighborhood exposure characteristics			
Average daily vehicular traffic volume (>10,000 vehicles/day)	26287 ± 15608	30704 ± 16303	24918 ± 16070^**^
Neighborhood background PM_2.5_^+^			
Annual 1-year average	18.0 ± 4.9	17.5 ± 4.3	14.7 ± 4.4^***^
Annual 5-year average	18.9 ± 4.0	18.2 ± 3.5	16.7 ± 3.3^***^
Neighborhood background ozone			
Annual 1-year average	24.2 ± 1.7	24.9 ± 1.9	24.5 ± 1.7
Annual 5-year average	23.9 ± 0.4	24.0 ± 0.6	23.8 ± 0.4

Air pollutant-emitting industries in neighborhood			
Charcoal burning food vendor	79 (62.7)	50 (54.9)	39 (55.7)
Bottled liquefied petroleum gas (LPG) depository	64 (50.8)	41 (45.1)	31 (44.3)
Restaurants	54 (42.9)	30 (33.0)	23 (32.9)
Bakeries	83 (65.9)	48 (52.7)	36 (51.4)
Gasoline station	22 (17.5)	13 (14.3)	11 (15.7)
Mechanic shops	42 (33.3)	19 (20.9)	19 (27.1)
Metalworking shop	9 (7.1)	10 (11.0)	5 (7.1)
Furniture/woodworking shop	7 (5.6)	3 (3.3)	3 (4.3)
Textile factory	4 (3.2)	2 (2.2)	5 (7.1)

^+^Estimated PM_2.5_.

^*^ *P* = 0.02; ^**^ *P* = 0.046; ^***^ *P* = 0.0001.

**Table 3:** Association of traffic indicators with carotid intima-media thickness (cIMT) measurements in child participants (*n* = 287).

	Mean difference (95% CI)	Mean difference (95% CI)	Mean difference (95% CI)
	Residential distance to traffic		
	<100 meters	100–199 meters	≥200 meters
cIMT mean (*μ*m)			
Unadjusted model	6.0 (4.1, 7.9)^*^	1.7 (0.4, 3.7)	Ref. category
Model 1^a^	5.9 (3.9, 7.8)^*^	1.2 (0.8, 3.3)	Ref. category
Model 2^b^	5.7 (3.7, 7.8)^*^	1.4 (0.7, 3.4)	Ref. category
cIMT maximum (*μ*m)			
Unadjusted model	6.0 (4.2, 8.2)^*^	1.1 (1.0, 3.2)	Ref. category
Model 1^a^	6.1 (4.1, 8.1)^*^	0.8 (1.3, 3.0)	Ref. category
Model 2^b^	5.9 (3.8, 7.9)^*^	0.9 (1.3, 0.3)	Ref. category

	Distance-weighted traffic density (DWTD)^c^		
	Tertile 3	Tertile 2	Tertile 1

cIMT mean (*μ*m)			
Unadjusted model	4.8 (2.9, 6.7)^*^	3.6 (1.7, −5.5)	Ref. category
Model 1^a^	4.8 (2.8, 6.7)^**^	3.4 (1.4, −5.4)	Ref. category
Model 2^b^	4.8 (2.8, 6.7)^*^	3.4 (1.5, −5.4)	Ref. category
cIMT maximum (*μ*m)			
Unadjusted model	5.2 (3.2, 7.2)^*^	3.4 (1.4, −5.4)	Ref. category
Model 1^a^	5.1 (3.1, 7.0)^*^	3.1 (1.1, −5.2)	Ref. category
Model 2^b^	5.0 (3.0, 7.0)^*^	3.0 (1.0, −5.1)	Ref. category

^a^Model 1: estimate adjusted for factors associated with cIMT and atherogenesis in healthy children: age, sex, BMI, mean arterial pressure, fasting total blood cholesterol, positive family CVD history, and systemic inflammation markers (hsCRP, IL-6).

^b^Model 2: estimate adjusted for annual neighborhood background concentrations of PM_2.5_ and ozone, residence altitude, average time spent outdoors (≥240 minutes/day versus <240 minutes/day), average time residence outside windows opened during day (≥60 minutes/day versus <60 minutes/day), and presence of outside smoker in household.

^c^Distance-weighted traffic density: Tertile 3: 10,128–65,770, Tertile 2: 783–9,876, and Tertile 1: 0–76.

^*^ *P* = 0.0001; ^**^ *P* = 0.001.
